# Concurrent chemotherapy with partial breast irradiation in triple negative breast cancer patients may improve disease control compared with sequential therapy

**DOI:** 10.3389/fonc.2023.1146754

**Published:** 2023-07-12

**Authors:** Ryan Rhome, Jean Wright, Lana De Souza Lawrence, Vered Stearns, Antonio Wolff, Richard Zellars

**Affiliations:** ^1^ Department of Radiation Oncology, Indiana University Hospital, Indianapolis, IN, United States; ^2^ Department of Radiation Oncology, The Johns Hopkins Hospital, Johns Hopkins Medicine, Baltimore, MD, United States; ^3^ Christiana Care Health System, Wilmington, DE, United States; ^4^ Department of Oncology, Division of Women’s Malignancies, The Johns Hopkins Hospital, Johns Hopkins Medicine, Baltimore, MD, United States

**Keywords:** partial breast irradiation, triple negative breast cancer, concurrent chemoradiation, breast cancer, clinical trial

## Abstract

**Purpose:**

To report outcomes on a subset of patients with triple negative breast cancer (TNBC) treated on prospective trials with post-lumpectomy partial breast irradiation and concurrent chemotherapy (PBICC) and compare them to a retrospectively assessed similar cohort treated with whole breast irradiation after adjuvant chemotherapy (WBIaC).

**Methods and materials:**

Women with T1-2, N0-1 invasive breast cancer with ≥ 2mm lumpectomy margins were offered therapy on one of two PBICC trials. PBI consisted of 40.5 Gy in 15 daily 2.7 Gy fractions delivered concurrently with the first 2 cycles of adjuvant chemotherapy. The comparison cohort received WBI to a median dose of 60.7 Gy, (including boost, range 42.5 – 66 Gy), after completion of non-concurrent, adjuvant chemotherapy. We evaluated disease-free survival (DFS), and local/loco-regional/distant recurrence-free survival (RFS). We compared survival rates using Kaplan-Meier curves and log-rank test of statistical significance.

**Results:**

Nineteen patients with TNBC were treated with PBICC on prospective protocol, and 49 received WBIaC. At a median follow-up of 35.5 months (range 4.8-71.9), we observed no deaths in the PBICC cohort and 2 deaths in the WBIaC cohort (one from disease recurrence). With a median time of 23.4 (range 4.8 to 47) months, there were 7 recurrences (1 nodal, 4 local, 4 distant), all in the WBIaC group. At 5 years, there was a trend towards increased local RFS (100% vs. 85.4%, *p=*0.17) and loco-regional RFS (100% vs. 83.5, *p*=0.13) favoring the PBICC cohort. There was no significant difference in distant RFS between the two groups (100% vs. 94.4%, *p*=0.36). Five-year DFS was 100% with PBICC vs.78.9% (95% CI: 63.2 to 94.6%, *p*=0.08) with WBIaC.

**Conclusion:**

This study suggests that PBICC may offer similar and possibly better outcomes in patients with TNBC compared to a retrospective cohort treated with WBIaC. This observation is hypothesis-generating for prospective trials.

## Introduction

Triple negative breast cancer (TNBC) is characterized by lack of expression of estrogen (ER) and progesterone (PR) receptors and lack of overexpression of the human epidermal growth factor receptor 2 (HER2). Women with TNBC are reported to have inferior overall survival, disease free survival, and local control than their non-TNBC counterparts when treated with whole breast irradiation (WBI) (1–3).

Routine management of stage I and II TNBC usually includes mastectomy or breast conserving surgery (BCS) followed by sequential administration of chemotherapy and 3 to 6 weeks of daily WBI (with length of course predicated on nodal coverage, fractionation scheme, and use of boost) (4). In this regard, concurrent chemotherapy and radiation offers potential logistic benefits. While shortening the overall duration of therapy, both adjuvant treatments are completed sooner after surgery. Concurrency can also take advantage of potential oncologic synergy between the two modalities in improving tumor control. Concurrent chemoradiation is used in most other adenocarcinoma-based disease sites, including lung, gastrointestinal, and bladder cancers (5–9), albeit often in the definitive or pre-operative setting. However, concerns of prohibitive toxicity with concurrent administration of anthracycline-based chemotherapy regimens and others along with whole breast radiotherapy have made this approach unpopular (10). The smaller fields employed during partial breast irradiation potentially allow for mitigation of this concurrent toxicity and acceleration of the radiotherapy schedule.

We previously reported results of the first of two prospective phase I/II trials of PBI and concurrent chemotherapy (PBICC) in women with early stage breast cancer (11). Given reports of inferior oncologic outcomes in patients with TNBC and the potential of improved local control with concurrent chemotherapy and irradiation, we hypothesized that patients with TNBC treated with our novel PBICC approach will have similar or improved clinical outcomes as TNBC patients treated more traditionally with WBI after chemotherapy (WBIaC). In this report, we describe the outcome of the subset of TNBC patients enrolled in these PBICC trials. To provide an internal contemporary reference, we also retrospectively describe the outcomes of a series of patients with TNBC patients treated with WBIaC during the same time period.

## Materials and methods

### Study participants

We evaluated a subgroup of 19 TNBC patients treated on two prospective trials of PBICC that enrolled women with T1-2, N0-1 invasive breast cancer and ≥ 2mm lumpectomy margins between 2004-2009. Both trials were approved by the Institutional Review Board. We also retrospectively identified 51 similar patients with TNBC (T1-2, N0-1 invasive breast cancer with ≥ 2mm lumpectomy margins), treated with standard WBIaC followed by standard chemotherapy at Johns Hopkins University between 2004 and 2009 by using an Institutional Review Board-approved database and chart review. Full details on study designs and participants can be found in the original publication (11).

### Radiotherapy

All patients underwent three-dimensional conformal or intensity-modulated radiation treatment planning, using five to seven non-coplanar photon beams.

### WBIaC

The median dose of WBI (including boost) in the triple-negative comparison cohort was 59.89 Gy (range 42.56 – 66.60 Gy). Whole breast radiotherapy was delivered in 180-270 cGy fractions. Nodal regions were treated in 20% of the WBIaC patients.

### PBICC

PBI consisted of 40.5 Gy in 15 daily 2.7 Gy fractions delivered concurrently with the first 2 of 4 cycles of chemotherapy. For the PBI trials, the clinical target volume (CTV) was defined by a uniform expansion on the lumpectomy cavity, as delineated on computed tomography (CT), by 1.5cm in all directions then cropped to 5mm from skin surface and the chest wall/lung interface. The planning target volume (PTV) was created by uniformly expanding the CTV by 5 mm. Nodal regions did not receive directed radiotherapeutic treatment.

### Chemotherapy

WBIaC and PBICC patients received cyclophosphamide +doxorubicin +/- paclitaxel (AC+T) or cyclophosphamide + docetaxel (TC), at the discretion of the treating medical oncologist.

In all WBIaC cases, radiotherapy was delivered after adjuvant chemotherapy. Decisions about additional systemic chemotherapy after completion of PBICC were made independently by the medical oncologist and the patient.

### Endpoints and statistical analysis

Two patients who were lost to follow-up within 12 months of lumpectomy were excluded from the retrospective cohort, therefore 49 patients were considered evaluable. Primary endpoints were disease-free survival (DFS), local recurrence-free survival (RFS), locoregional RFS, and distant RFS which were measured from the date of lumpectomy to time of any recurrence, local failure, locoregional failure, or distant failure, respectively. Local failure was defined as a biopsy-proven recurrence in the ipsilateral breast. Locoregional failure was defined as recurrence either in the ipsilateral breast or regional nodes, including the axilla, internal mammary nodes, or supraclavicular nodes. Distant failure was defined as the development of metastatic foci other than regional lymph nodes. Only distant recurrences that occurred as a first recurrence were considered in the estimation of distant disease-free survival. Progression free survival (PFS) curves comparing treatment modalities were analyzed using the Kaplan-Meier method, and comparisons were made using log-rank χ^2^ testing.

Fisher’s exact and χ^2^ tests were used to compare proportions between two or more groups. Nonparametric data testing consisted of the Mann-Whitney *U* test and the Kruskal-Wallis nonparametric analysis of variance test for comparison of two and three different groups. All statistics were calculated with SSPS (19.0 for Windows; SSPS, Inc., Chicago, Illinois, USA) and GraphPad Prism (5.0 for Windows; GraphPad Software Inc) software. A two-tailed *P* value of < 0.05 was considered statistically significant for all analyses.

## Results

### Patient characteristics

Patient characteristics are summarized in **Table 1**. The median follow-up was 33.9 (range 4.8 to 71.9) and 41.9 (range 17 to 68.4) months for the WBIaC and PBICC groups respectively. Median follow up time for all patients was 35.5 months (range 4.8 to 71.9).

**Table 1:** Patient Characteristics.

Patient Characteristics		Triple Receptor Negative		
		WBI-SC n=49	PBI-CC *n=* 19	p
Median Age (range)		54 (36-80)	61 (40-75)	0.34
cT stage	T1	31 (63%)	11 (58%)	0.68
	T2	18 (37%)	8 (42%)	
cN stage	N0	36 (73%)	15 (79%)	0.64
	N1	13 (27%)	4 (21%)	
Menopausal Status	Pre-	16 (33%)	5 (26%)	0.61
	Post-	33 (67%)	14 (74%)	
Race	Caucasian	23 (47%)	8 (42%)	0.90
	African-American	20 (41%)	8 (42%)	
	Other/ Not specified	6 (12%)	3 (16%)	
Treatment				
Chemotherapy	AC	10 (20%)	6 (32%)	0.21
	AC+P	31 (64%)	9 (47%)	
	TC	8 (16%)	4 (21%)	
Median total RT dose incl. boost (cGy) (Range)		5989 (4256-6660)	4050 (4050-4050)	
Median RT dose per fraction (cGy) (Range)		204 (180-266)	270 (270-270)	
Pathologic Characteristics				
Mean Primary Tumor size (cm)		1.98 (SD 0.91)	1.82 (SD 0.83)	0.65
Median Number of Nodes Examined (Range)		3 (1-28)	4 (1-22)	0.47
Median Number of Nodes Positive (Range)		0 (0-4)	0 (0-2)	0.59
LVI	Present	5 (10%)	3 (16%)	0.48
	Absent	38 (78%)	12 (63%)	
	Unknown	6 (12%)	4 (21%)	
Extent of DCIS	<40%	48 (98%)	18 (95%)	0.48
	≥40%	1 (2%)	1 (5%)	

AC, doxorubicin + cyclophosphamide; AC+P, doxorubicin + cyclophosphamide followed by paclitaxel; TC, cyclophosphamide + docetaxel; LVI, lymphovascular invasion; DCIS, ductal carcinoma in situ.

There was no statistically significant difference between the WBIaC and PBICC groups with respect to clinical T stage, clinical N stage, median age, race, menopausal status, type of chemotherapy used, or pathologic features.

### Triple negative breast cancer outcomes

Overall, seven of 49 (14.3%) of TNBC patients treated with WBIaC had disease recurrence at a median of 23.4 (range 4.8 to 47) months. Sites of recurrence included one nodal, four local, and two distant. Two WBIaC patients died (one of disease recurrence). There were no deaths or recurrences in the PBICC cohort. Patterns of treatment are summarized in **Table 2**.

**Table 2:** Patterns of treatment Failure in Breast Cancer Patients with Triple Negative Receptor Status According to Treatment Modality.

	WBI-SC	PBI-CC	p Value
5 yr Disease-free Survival % (95% CI)	78.9 % (63.2 to 94.6%)	100%	0.08
5 yr Local Recurrence % (95% CI)	14.6 % (0.0 to 29.5% )	0%	0.17
5 yr Locoregional Recurrence % (95% CI)	16.5 % (1.4 to 31.6%)	0%	0.13
5 yr Distant Metastasis % (95% CI)	5.6% (-2.2 to 13.4%)	0%	0.36

### Local recurrence

**Figure 1:**
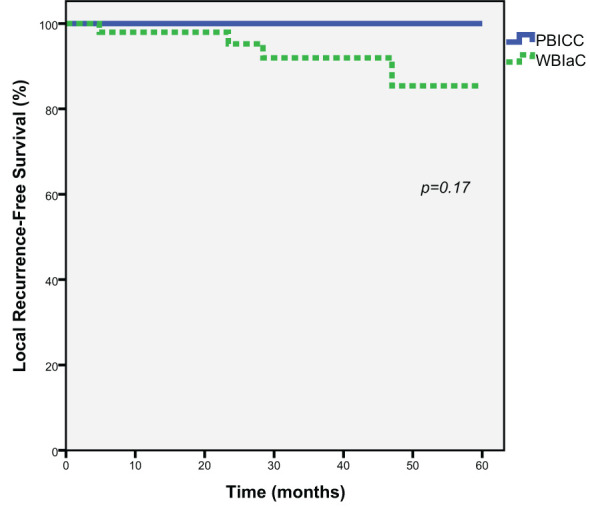
Kaplan-Meier estimates of Local Progression-Free Survival for Triple Negative Receptor Patients treated with PBICC and WBIaC.

At 5 years, there was a numeric trend towards decreased local recurrence (0% vs. 14.6%, *p=*0.17) in the PBICC cohort compared to the WBIaC cohort. The 3 year rates of local recurrence were 0% and 7.9% for PIBCC and WBIaC cohorts, respectively. **Figure 1** demonstrates Kaplan-Meier analysis comparing both groups with respect to local recurrence-free survival. The median time to initial-site local recurrence was 25.9 months (range 4.8 to 47).

### Locoregional recurrence

**Figure 2:**
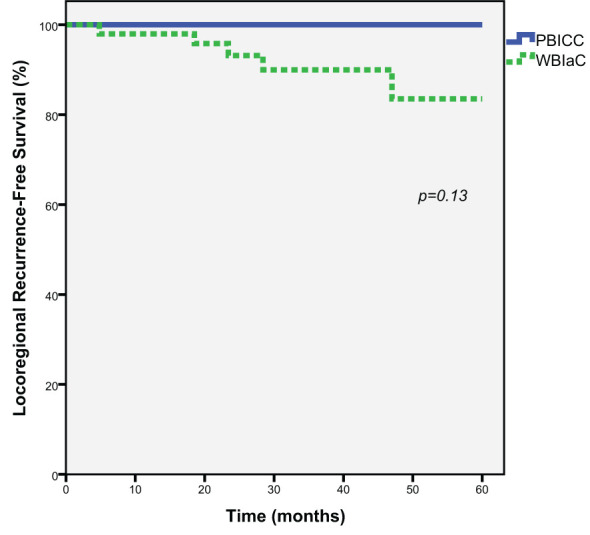
Kaplan-Meier estimates of Locoregional Progression-Free Survival for Triple Negative Receptor Patients treated with PBICC and WBIaC.

At 5 years, there was a trend towards decreased loco-regional recurrence (0% vs. 17.8%, *p*=0.13) in the PBICC cohort compared to the WBIaC cohort. The 3 year locoregional recurrence rates were 0% and 13.2% for the PBICC and WBIaC cohorts, respectively. **Figure 2** demonstrates Kaplan-Meier analysis comparing both groups with respect to locoregional recurrence-free survival. The time to recurrence in the single patient with initial-site nodal recurrence was 18.5 months. The median time to any locoregional recurrence was 23.4 months (range 4.8 to 47 months).

### Distant recurrence

**Figure 3:**
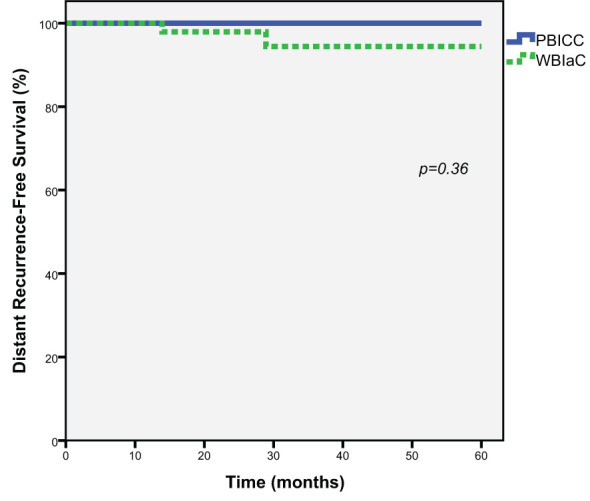
Kaplan-Meier estimates of Distant Progression-Free Survival for Triple Negative Receptor Patients treated with PBICC and WBIaC.

At 3 and 5 years, there was a no significant difference in the rate of distant metastasis (0% vs. 5.6%, *p*=0.36) between the PBICC and WBIaC cohorts. **Figure 3** demonstrates Kaplan-Meier analysis comparing both groups with respect to distant recurrence-free survival. The median time to initial-site distant recurrence was 21.4 months (range 13.9 to 28.9).

### Disease-free survival

**Figure 4:**
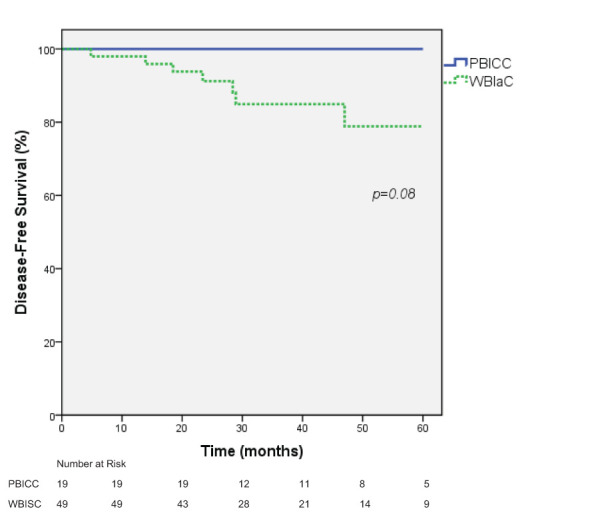
Kaplan-Meier estimates of Disease-Free survival for Triple Negative Receptor Patients treated with PBICC and WBIaC.

Five-year DFS estimates were 78.9% (95% CI: 63.2 to 94.6%) vs. 100% in the WBIaC vs. PBICC group respectively by Kaplan-Meier survival analysis, p=0.08 (**Figure 4**). The 3 year DFS for the groups was 83.6% in the WBIaC group and 100% for the PBICC group. The hazard ratio for disease-free survival was 0.24, numerically in favor of the PBICC group at 5 years (95% CI: 0.05 to 1.12).

## Discussion

Patients with TNBC are at increased risk of breast cancer recurrence. Radiation with concurrent chemotherapy is known to improve local control *via* the radiation sensitizing effects of chemotherapy in many other disease sites (5–9). We chose to retrospectively review TNBC patients treated on 2 prospective phase I/II trials of PBI and concurrent chemotherapy, and compare their outcomes to retrospectively reviewed TNBC patients treated with WBI after adjuvant chemotherapy during the same period. The results of this study showed a trend towards improved local and loco-regional recurrence-free survival and overall disease-free survival with PBI and concurrent chemotherapy. As previously reported from the entire phase I/II cohort, this approach also has a favorable safety profile (11) in contrast with some other reports of concurrent chemoradiation for breast cancer (10). Specifically, patients in the entire cohort had an 84% rate of grade 1 dermatitis and 0% rate of grade 2+ skin toxicity. There were no incidences of pneumonitis (0%) in that report.

Increased local recurrences in women with TNBC treated with lumpectomy and whole breast irradiation are noted in several reports. In a paper by Arvold et al. (12), 1434 patients treated with breast-conserving therapy were divided into standard breast cancer sub-types, 171 of whom had TNBC. With a median follow-up of 84 months, the authors reported that the TNBC sub-type was independently associated with increased local recurrence on multivariate analysis (MVA). Zaky et al. (13) reviewed 193 and 160 women with TNBC and non-TNBC respectively, all treated with BCS and WBI. With a median follow-up of 3.4 yrs, the authors reported a 12% and 4% rate of local recurrence respectively (p=0.01). On MVA, TNBC was again independently associated with local recurrence. This elevated rate of local recurrence has also been reported in patients treated with PBI. Pashtan et al. (14) recently reported a 5 year actuarial local recurrence rate of 32.5% in 9 TNBC patients treated with 3D-Accelerated partial breast irradiation. When compared to HR positive/Her 2 negative patients, TNBC patients treated with PBI had a local recurrence hazard ratio of 15.2 (95% CI, 2.5-91). However, this increased rate of local recurrence after breast conserving therapy in TNBC patients is not a universal finding. A study by Wilkinson et al. (15), which included 20 TNBC patients and 182 receptor positive patients (almost half of whom were treated with 3D-APBI), reported a 0% actuarial rate of ipsilateral breast recurrence, nodal recurrence and distant metastases at 5 years in the TNBC cohort, which was not statistically distinct from the receptor positive patients.

There is also a suggestion of worse outcomes in patients with TNBC with regards to regional control, distant metastasis, and survival. For example, Haffty et al. (16) reported a statistically significant inferior nodal relapse-free rate (94 vs 99%), distant metastasis-free rate (68% vs. 83%) and cause-specific survival (72 vs. 85%) at 5 years with conventional breast-conserving therapy for TNBC patients compared to others. Wilder et al. (17) also demonstrated significantly inferior non-local relapse (81 vs 100%) and cause-specific survival (89 vs 100%) at 3 years for TNBC patients compared to others, when treated with PBI. Taken together with the previous discussion of local recurrences, TNBC patients are at higher risk for both local and distant recurrences.

These local and distant recurrence issues may have different solutions. One implication of the increased recurrences seen with TNBC is a relative radioresistance of this phenotype. Concurrent chemotherapy has been shown to improve response rates and overcome radioresistance to certain degrees in multiple tumor types (9). The trend towards improved outcomes with PBICC compared to WBIaC in our study may be due to the radiation sensitizing effects of concurrent chemotherapy but may also be due to the temporal proximity of radiation to surgery. Most commonly, chemotherapy is delivered after surgery and before radiation. Consequently, chemotherapy delays the start of radiation therapy. The importance of RT timing following surgery to reduce the risk of local recurrence is controversial. Bellon et al. (4) randomized 244 women to receive 12 weeks of cyclophosphamide, doxorubicin, methotrexate, fluorouracil and prednisone (CAMFP) before or after RT. At a median follow-up of 135 months, there were no significant differences between the chemotherapy-first and radiotherapy-first arms in time to any event, distant metastasis or death. Conversely, a systematic review by Huang et al. (18) of 11 studies involving 1,927 breast cancer patients demonstrated an increase in the 5-year locoregional recurrence from 6% in the RT-first group to 16% in the chemotherapy-first group (HR 2.28, 95% CI, 1.45 to 3.57). Additional evidence may possibly be seen in the study of PBI by Pashtan et al. above, in which all TNBC PBI recurrences occurred in patients who had their radiation delayed secondary to chemotherapy. How this timing is affected by the increasing use of hypofractionated (19, 20) and ultrahypofractionated (21, 22) radiation therapy is unknown. By definition, the PBICC strategy described here has a shorter interval between surgery and radiation, as the concurrent chemoradiation starts after the patient is sufficiently healed from surgery whereas the conventional standard is to complete all of adjuvant chemotherapy (several months of therapy) prior to radiation.

Our study suggests that combining concurrent chemotherapy with radiation may improve outcomes in TNBC. Concurrency in breast cancer has traditionally been avoided due to previous reports of increased toxicity (10). A recent Phase I prospective trial of concurrent carboplatin with whole breast standard fractionation radiation therapy, and found favorable safety profiles, with planned Phase II study opening thereafter (23). The patients in these trials had multiagent regimens consistent with standard recommendations for TNBC such as AC+T or TC.

We posit that the use of PBI with concurrent chemotherapy would mitigate these toxicities, and that has been supported by previous reports of these trials (11). Nonetheless, there is enough uncertainty about their propensity to recur more often, that the ASTRO guidelines for PBI stress caution in patients who are hormone-receptor-negative (24, 25). Part of the rationale in the discussion for those guidelines was a relative paucity of TNBC patients on APBI clinical trials, rather than specifically citing the recurrence propensity. For example, NSABP B39 and Florence trials had 19% and 1-2.3% of triple negative patients, respectively (22, 26). RAPID and IMPORT LOW studies had only 9-11% and 5% of ER negative patients, respectively (27, 28). One study has suggested ER negativity as a predictor of local recurrence in brachytherapy APBI (29). In contrast, Goulding et al. (30) analyzed patients on two prospective APBI trials that were treated with external beam RT, and when specifically looking at TNBC and other “high risk” patients compared with “suitable” patients, TNBC was not associated with higher in-breast recurrence risk, with no local recurrences occurring in this cohort.

There are limitations to this analysis. Although the patients treated with PBICC were participants of two prospective clinical trials, the trials were not originally designed to address this question. Thus our reported analyses of both PBICC and WBIaC cohorts are truly retrospective in nature. As a retrospective study, it is subject to limitations common with this type of analysis. Specifically there may be patient and treatment differences as well as unknown factors that may have influenced the results. We attempted to mitigate these limitations by choosing comparative cohort (WBIaC) patients with comparable stages and treated during the same time period. For instance, the doses of radiation used in the WBIaC patients were more variable and with a higher range than the PBICC group. While this is an imbalance, it does further support the trend toward control in triple negative patients with concurrent chemotherapy even at lower overall radiation doses in the concurrent cohort. An additional limitation is that our study cohorts are relatively small, likely explaining the lack of statistical significance in our findings. Nonetheless, the local recurrence rate in the WBIaC cohort is comparable to other published studies. For example, Dent et al. (3) reported a 13% rate of local recurrence in 180 TNBC patients with clinically localized disease treated with WBIaC, with a mean time to local recurrence of 2.8 years. Conversely, the lack of local recurrences in the PBICC cohort is unexpected. As the risk of recurrence in TNBC rapidly declines after the first 3 years, we believe that the median follow-up of the TNBC patients in our study is likely to be adequate to capture a majority of recurrence events. While small, the PBICC cohort is, to the best of our knowledge, the first report of breast cancer outcomes using this approach in TNBC from prospectively collected data. Given these limitations, we consider our results hypothesis-generating.

## Conclusion

The finding of extremely low recurrence rates in patients with TNBC treated with PBICC differs both from the comparison cohort of retrospectively reviewed contemporary patients treated with WBIaC, and from earlier reports of a high rate of local recurrence in TNBC patients treated with PBI. This data generates a hypothesis that the PBICC approach is associated with improved clinical outcomes, potentially due to shorter intervals from surgery to radiotherapy and/or to a synergy between radiotherapy and chemotherapy. The ongoing randomized Phase II trial (PBI 3.0, NCT01928589) currently accruing patients that will provide additional information on outcomes using PBICC.

## Data availability statement

The raw data supporting the conclusions of this article will be made available by the authors, without undue reservation.

## Ethics statement

The studies involving human participants were reviewed and approved by Johns Hopkins Institutional Review Board. The patients/participants provided their written informed consent to participate in this study.

## Author contributions

RR: Manuscript creation, data interpretation and analysis. JW: Data interpretation and analysis, review/editing manuscript. LD: Data interpretation and analysis, review/editing manuscript. VS: Patient enrollment, review/editing manuscript, data interpretation. AW: Patient enrollment, review/editing manuscript, data interpretation. RZ: Trial design and execution, data collection, and review/editing manuscript. All authors contributed to the article and approved the submitted version.
